# (1β,2α,3α,7α,11α,13β)-1,3,7,11-Tetra­acet­oxy-2,13-bis­(benz­yloxy)-21-methyl-19,21-secohetisan-19-al hemi­hydrate

**DOI:** 10.1107/S1600536809018315

**Published:** 2009-05-29

**Authors:** Feng-Zheng Chen, Qing-Xiang Xiang, Yuan-Qin Zhang, Jun-Ru Xiong

**Affiliations:** a Department of Chemistry and Life Sciences, Leshan Teachers’ College, Leshan 614004, People’s Republic of China

## Abstract

In the crystal structure of the title compound, C_43_H_46_NO_13_·0.5H_2_O, the mol­ecule assumes a U-shaped conformation, the terminal benzene rings being approximately parallel and partially overlapped with each other. The mol­ecule contains eight alicyclic and heterocyclic rings. The cyclo­hexane rings adopt chair conformations, the other three six-membered carbocyclic rings form a bicyclo­[2.2.2]octane system with a boat conformation for each six-membered ring, the six-membered heterocyclic ring has a chair conformation and both of the five-membered rings have envelope conformations. The solvent water mol­ecule links with the organic mol­ecule *via* classic O—H⋯O and weak C—H⋯O hydrogen bonding in the crystal structure.

## Related literature

For general background, see: Deng *et al.* (1992 ▶).
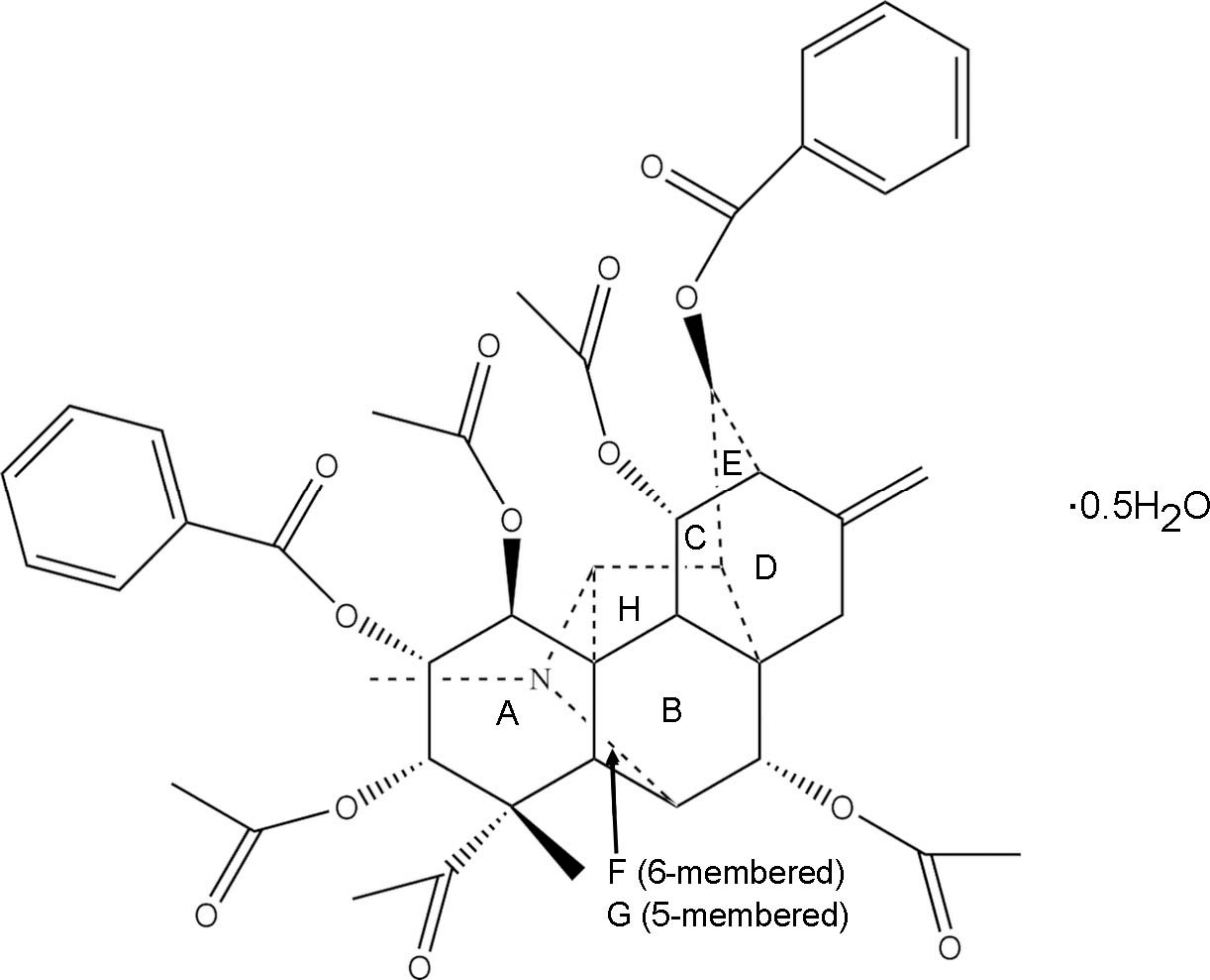


## Experimental

### 

#### Crystal data

C_43_H_46_NO_13_·0.5H_2_O
*M* *_r_* = 793.82
Orthorhombic, 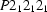

*a* = 11.517 (3) Å
*b* = 18.045 (4) Å
*c* = 19.241 (4) Å
*V* = 3998.7 (16) Å^3^
*Z* = 4
Mo *K*α radiation
μ = 0.10 mm^−1^
*T* = 292 K
0.52 × 0.46 × 0.42 mm

#### Data collection

Enraf–Nonius CAD-4 diffractometer
Absorption correction: none
4333 measured reflections
4092 independent reflections
2220 reflections with *I* > 2σ(*I*)
*R*_int_ = 0.007
3 standard reflections every 250 reflections intensity decay: 2.0%

#### Refinement

*R*[*F* ^2^ > 2σ(*F* ^2^)] = 0.052
*wR*(*F* ^2^) = 0.150
*S* = 1.00
4092 reflections
530 parameters
H-atom parameters constrained
Δρ_max_ = 0.21 e Å^−3^
Δρ_min_ = −0.24 e Å^−3^

### 

Data collection: *DIFRAC* (Gabe & White, 1993 ▶); cell refinement: *DIFRAC*; data reduction: *NRCVAX* (Gabe *et al.*, 1989 ▶); program(s) used to solve structure: *SHELXS97* (Sheldrick, 2008 ▶); program(s) used to refine structure: *SHELXL97* (Sheldrick, 2008 ▶); molecular graphics: *ORTEP-3 for Windows* (Farrugia, 1997 ▶); software used to prepare material for publication: *WinGX* (Farrugia, 1999 ▶).

## Supplementary Material

Crystal structure: contains datablocks I, global. DOI: 10.1107/S1600536809018315/xu2510sup1.cif

Structure factors: contains datablocks I. DOI: 10.1107/S1600536809018315/xu2510Isup2.hkl

Additional supplementary materials:  crystallographic information; 3D view; checkCIF report

## Figures and Tables

**Table 1:** Hydrogen-bond geometry (Å, °)

*D*—H⋯*A*	*D*—H	H⋯*A*	*D*⋯*A*	*D*—H⋯*A*
O1*W*—H1*A*⋯O11^i^	0.89	2.38	3.205 (15)	155
O1*W*—H1*B*⋯O6	0.90	2.39	3.208 (15)	151
C15—H15*A*⋯O4^ii^	0.97	2.48	3.359 (8)	150
C23—H23*B*⋯O1*W*	0.96	2.58	3.491 (17)	159
C29—H29⋯O7^iii^	0.93	2.40	3.290 (8)	160

Symmetry codes: (i) 
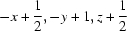
; (ii) 
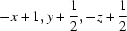
; (iii) 
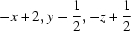
.

**Fig. 1.**
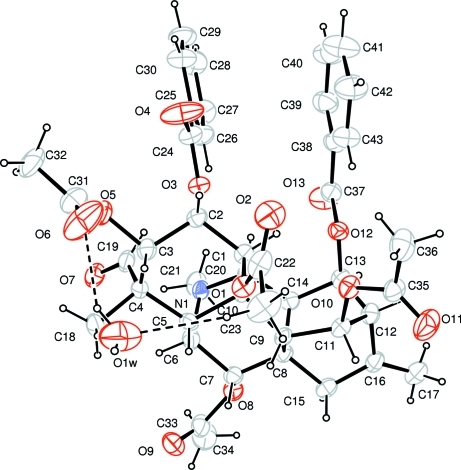
The molecular structure of the title compound. Displacement ellipsoids are drawn at the 30% probability level. Dashed line indicates hydrogen bonding.

## supplementary crystallographic information

### Comment

The diterpenoid alkaloid, acetyldelgradine, has been isolated from *Delphiniumgrandiflorum L.* (Deng *et al.*, 1992) and its structure was established from the spectroscopic data. In our current investigation, the title compound was isolated from *Aconitum carmichaeli Debx*, and its structure was confirmed by X-ray diffraction study.

Themolecular structure of the title compound is shown in Fig. 1. The molecule of the title compound assumes an U-shaped conformation, with terminal benzene rings being approximately parallel and partially overlapped to each other. The molecule contains eight alicyclic and heterocyclic rings. Cyclohexane rings A (C1/C2/C3/C4/C5/C10) and B (C5/C6/C7/C8/C9/C10) adopt chair conformations; six-membered rings C (C8/C9/C11/C12/C13/C14), D (C8/C9/C11/C12/C15/C16) and E (C8/C12/C13/C14/C15/C16) form a bicycle [2.2.2] octane system with the boat conformation for each six-membered ring C, D and E; the six-membered heterocyclic ring F (C6/C7/C8/C14/C20/N1) adopts a screw-boat conformation; while the five-membered rings G (C5/C6/C10/C20/N1) and H (C8/C9/C10/C14/C20) adopt the same envelope conformation.

The lattice water molecule links with the organic molecule *via* O—H···O hydrogen bonding, and weak molecular C—H···O hydrogen bonding is also present in the crystal structure (Table 1).

### Experimental

The title compound was isolated from the roots of Aconitum carmichaeli Debx and crystals suitable for X-ray structure analysis were obtained by slow evaporation from an acetone-water solution at room temperature.

### Refinement

Water H atoms were placed on chemical-sensible positions, other H atoms were located geometrically with C—H = 0.93–0.98 Å. H atoms were refined using a riding model with *U*_iso_(H) =1.2*U*_eq_(C) and *U*_iso_(H) = 1.5*U*_eq_(O). The absolute configuration was not determined, and Friedel pairs were merged.

### Crystal data

**Table:**

C_43_H_46_NO_13_·0.5H_2_O	*F*(000) = 1680
*M**_r_* = 793.82	*D*_x_ = 1.319 Mg m^−^^3^
Orthorhombic, *P*2_1_2_1_2_1_	Mo *K*α radiation, λ = 0.71073 Å
Hall symbol: P 2ac 2ab	Cell parameters from 36 reflections
*a* = 11.517 (3) Å	θ = 4.6–9.6°
*b* = 18.045 (4) Å	µ = 0.10 mm^−^^1^
*c* = 19.241 (4) Å	*T* = 292 K
*V* = 3998.7 (16) Å^3^	Block, colourless
*Z* = 4	0.52 × 0.46 × 0.42 mm

### Data collection

**Table:**

Enraf–Nonius CAD-4 diffractometer	*R*_int_ = 0.007
Radiation source: fine-focus sealed tube	θ_max_ = 25.5°, θ_min_ = 1.6°
graphite	*h* = −3→13
ω/2θ scans	*k* = −8→21
4333 measured reflections	*l* = −4→23
4092 independent reflections	3 standard reflections every 250 reflections
2220 reflections with *I* > 2σ(*I*)	intensity decay: 2.0%

### Refinement

**Table:**

Refinement on *F*^2^	Primary atom site location: structure-invariant direct methods
Least-squares matrix: full	Secondary atom site location: difference Fourier map
*R*[*F*^2^ > 2σ(*F*^2^)] = 0.052	Hydrogen site location: inferred from neighbouring sites
*wR*(*F*^2^) = 0.150	H-atom parameters constrained
*S* = 1.00	*w* = 1/[σ^2^(*F*_o_^2^) + (0.0817*P*)^2^] where *P* = (*F*_o_^2^ + 2*F*_c_^2^)/3
4092 reflections	(Δ/σ)_max_ = 0.001
530 parameters	Δρ_max_ = 0.21 e Å^−^^3^
0 restraints	Δρ_min_ = −0.24 e Å^−^^3^

### Special details

**Table:**

Geometry. All esds (except the esd in the dihedral angle between two l.s. planes) are estimated using the full covariance matrix. The cell esds are taken into account individually in the estimation of esds in distances, angles and torsion angles; correlations between esds in cell parameters are only used when they are defined by crystal symmetry. An approximate (isotropic) treatment of cell esds is used for estimating esds involving l.s. planes.
Refinement. Refinement of *F*^2^ against ALL reflections. The weighted *R*-factor *wR* and goodness of fit *S* are based on *F*^2^, conventional *R*-factors *R* are based on *F*, with *F* set to zero for negative *F*^2^. The threshold expression of *F*^2^ > σ(*F*^2^) is used only for calculating *R*-factors(gt) *etc*. and is not relevant to the choice of reflections for refinement. *R*-factors based on *F*^2^ are statistically about twice as large as those based on *F*, and *R*- factors based on ALL data will be even larger.

### Fractional atomic coordinates and isotropic or equivalent isotropic displacement parameters (Å^2^)

**Table:**

	*x*	*y*	*z*	*U*_iso_*/*U*_eq_	Occ. (<1)
N1	0.8109 (4)	0.4658 (2)	0.2072 (2)	0.0424 (10)	
O1	0.4566 (3)	0.41227 (19)	0.30263 (17)	0.0473 (9)	
O2	0.3551 (4)	0.3061 (3)	0.3012 (3)	0.0824 (13)	
O3	0.6957 (3)	0.29181 (18)	0.25073 (15)	0.0438 (8)	
O4	0.6532 (5)	0.1788 (2)	0.2904 (3)	0.109 (2)	
O5	0.7455 (3)	0.3093 (2)	0.39340 (16)	0.0563 (10)	
O6	0.6180 (7)	0.2970 (4)	0.4783 (3)	0.137 (3)	
O7	0.9560 (4)	0.4295 (2)	0.3264 (2)	0.0677 (12)	
O8	0.7968 (4)	0.63060 (19)	0.16840 (17)	0.0547 (10)	
O9	0.8387 (5)	0.6961 (2)	0.2649 (2)	0.0863 (16)	
O10	0.3615 (3)	0.4339 (2)	0.16916 (18)	0.0506 (9)	
O11	0.2044 (4)	0.4848 (3)	0.1230 (3)	0.0971 (16)	
O12	0.5520 (3)	0.38202 (18)	0.07900 (15)	0.0492 (10)	
O13	0.7089 (5)	0.3518 (2)	0.0167 (2)	0.0816 (14)	
O1W	0.4527 (15)	0.4368 (6)	0.5068 (7)	0.157 (5)	0.50
H1A	0.3997	0.4441	0.5398	0.236*	0.50
H1B	0.5077	0.4021	0.5148	0.236*	0.50
C1	0.5433 (4)	0.3818 (3)	0.2552 (2)	0.0405 (12)	
H1	0.5055	0.3574	0.2157	0.049*	
C2	0.6125 (5)	0.3258 (3)	0.2978 (2)	0.0438 (13)	
H2	0.5599	0.2876	0.3158	0.053*	
C3	0.6726 (5)	0.3642 (3)	0.3580 (2)	0.0490 (15)	
H3	0.6127	0.3804	0.3908	0.059*	
C4	0.7491 (5)	0.4309 (3)	0.3412 (2)	0.0468 (14)	
C5	0.6813 (5)	0.4848 (3)	0.2924 (2)	0.0418 (13)	
H5	0.6320	0.5182	0.3197	0.050*	
C6	0.7700 (5)	0.5288 (3)	0.2504 (2)	0.0494 (14)	
H6	0.8321	0.5487	0.2799	0.059*	
C7	0.7118 (5)	0.5894 (3)	0.2074 (2)	0.0459 (13)	
H7	0.6728	0.6236	0.2393	0.055*	
C8	0.6210 (5)	0.5581 (3)	0.1574 (2)	0.0406 (12)	
C9	0.5323 (4)	0.5144 (3)	0.2020 (2)	0.0397 (12)	
H9	0.5078	0.5457	0.2409	0.048*	
C10	0.6107 (4)	0.4495 (2)	0.2311 (2)	0.0362 (12)	
C11	0.4261 (5)	0.5014 (3)	0.1534 (2)	0.0458 (13)	
H11	0.3729	0.5432	0.1599	0.055*	
C12	0.4632 (5)	0.5014 (3)	0.0769 (2)	0.0465 (14)	
H12	0.4015	0.4816	0.0471	0.056*	
C13	0.5756 (5)	0.4594 (3)	0.0665 (2)	0.0478 (14)	
H13	0.6009	0.4655	0.0182	0.057*	
C14	0.6697 (5)	0.4909 (3)	0.1149 (2)	0.0416 (13)	
H14	0.7379	0.5061	0.0880	0.050*	
C15	0.5672 (5)	0.6171 (3)	0.1110 (3)	0.0489 (14)	
H15A	0.5222	0.6513	0.1391	0.059*	
H15B	0.6280	0.6449	0.0879	0.059*	
C16	0.4908 (5)	0.5817 (3)	0.0584 (2)	0.0507 (15)	
C17	0.4573 (6)	0.6101 (3)	−0.0017 (3)	0.0678 (17)	
H17A	0.4830	0.6577	−0.0136	0.102*	
H17B	0.4097	0.5839	−0.0307	0.102*	
C18	0.7737 (6)	0.4725 (3)	0.4089 (2)	0.0660 (18)	
H18A	0.7038	0.4753	0.4360	0.099*	
H18B	0.8004	0.5216	0.3984	0.099*	
H18C	0.8323	0.4466	0.4348	0.099*	
C19	0.8629 (5)	0.4042 (3)	0.3101 (3)	0.0517 (15)	
H19	0.8607	0.3667	0.2771	0.062*	
C20	0.7053 (4)	0.4388 (3)	0.1735 (2)	0.0394 (12)	
H20	0.7120	0.3872	0.1581	0.047*	
C21	0.9154 (5)	0.4730 (4)	0.1657 (3)	0.0621 (16)	
H21A	0.9810	0.4798	0.1958	0.093*	
H21B	0.9081	0.5150	0.1354	0.093*	
H21C	0.9261	0.4290	0.1385	0.093*	
C22	0.3641 (6)	0.3712 (4)	0.3187 (3)	0.0628 (17)	
C23	0.2783 (6)	0.4138 (4)	0.3600 (4)	0.089 (2)	
H23A	0.2664	0.4614	0.3390	0.133*	
H23B	0.3069	0.4202	0.4065	0.133*	
H23C	0.2061	0.3872	0.3613	0.133*	
C24	0.7091 (5)	0.2182 (3)	0.2527 (3)	0.0515 (14)	
C25	0.8001 (5)	0.1921 (3)	0.2039 (3)	0.0517 (14)	
C26	0.8602 (6)	0.2382 (3)	0.1606 (3)	0.0606 (16)	
H26	0.8440	0.2887	0.1613	0.073*	
C27	0.9435 (6)	0.2121 (4)	0.1162 (3)	0.0739 (19)	
H27	0.9840	0.2442	0.0871	0.089*	
C28	0.9662 (7)	0.1361 (4)	0.1156 (4)	0.081 (2)	
H28	1.0205	0.1172	0.0845	0.097*	
C29	0.9110 (7)	0.0901 (4)	0.1591 (4)	0.082 (2)	
H29	0.9305	0.0401	0.1597	0.098*	
C30	0.8267 (6)	0.1156 (3)	0.2026 (4)	0.076 (2)	
H30	0.7867	0.0829	0.2313	0.091*	
C31	0.7080 (7)	0.2792 (4)	0.4532 (4)	0.0745 (19)	
C32	0.7891 (7)	0.2250 (4)	0.4806 (3)	0.092 (2)	
H32A	0.8670	0.2431	0.4752	0.138*	
H32B	0.7807	0.1792	0.4558	0.138*	
H32C	0.7733	0.2169	0.5290	0.138*	
C33	0.8555 (6)	0.6848 (3)	0.2056 (4)	0.0667 (18)	
C34	0.9414 (7)	0.7233 (4)	0.1601 (4)	0.099 (2)	
H34A	0.9086	0.7302	0.1147	0.148*	
H34B	1.0104	0.6937	0.1566	0.148*	
H34C	0.9604	0.7706	0.1798	0.148*	
C35	0.2509 (5)	0.4329 (3)	0.1512 (3)	0.0550 (15)	
C36	0.1945 (6)	0.3630 (4)	0.1650 (4)	0.086 (2)	
H36A	0.2522	0.3250	0.1705	0.130*	
H36B	0.1445	0.3506	0.1268	0.130*	
H36C	0.1494	0.3668	0.2067	0.130*	
C37	0.6245 (6)	0.3329 (3)	0.0482 (3)	0.0553 (15)	
C38	0.5864 (6)	0.2548 (3)	0.0582 (3)	0.0549 (16)	
C39	0.6559 (7)	0.2000 (3)	0.0315 (3)	0.076 (2)	
H39	0.7251	0.2121	0.0093	0.091*	
C40	0.6216 (10)	0.1249 (4)	0.0379 (4)	0.100 (3)	
H40	0.6682	0.0872	0.0204	0.120*	
C41	0.5203 (10)	0.1088 (5)	0.0698 (5)	0.115 (3)	
H41	0.4982	0.0594	0.0738	0.138*	
C42	0.4482 (8)	0.1630 (4)	0.0966 (4)	0.100 (2)	
H42	0.3782	0.1507	0.1178	0.120*	
C43	0.4837 (6)	0.2370 (3)	0.0908 (3)	0.0716 (19)	
H43	0.4374	0.2744	0.1092	0.086*	

### Atomic displacement parameters (Å^2^)

**Table:**

	*U*^11^	*U*^22^	*U*^33^	*U*^12^	*U*^13^	*U*^23^
N1	0.033 (2)	0.046 (2)	0.048 (2)	0.001 (2)	−0.006 (2)	0.001 (2)
O1	0.044 (2)	0.052 (2)	0.0460 (19)	0.003 (2)	0.0148 (19)	0.0070 (17)
O2	0.063 (3)	0.079 (3)	0.105 (3)	−0.011 (3)	0.009 (3)	0.012 (3)
O3	0.048 (2)	0.0353 (19)	0.0476 (18)	0.0034 (19)	0.0071 (18)	0.0019 (15)
O4	0.129 (5)	0.049 (3)	0.149 (4)	0.007 (3)	0.079 (4)	0.026 (3)
O5	0.062 (3)	0.061 (2)	0.0456 (19)	0.000 (2)	−0.010 (2)	0.0157 (18)
O6	0.158 (6)	0.148 (6)	0.105 (4)	0.059 (5)	0.057 (4)	0.077 (4)
O7	0.058 (3)	0.064 (3)	0.081 (3)	−0.003 (2)	−0.023 (2)	0.018 (2)
O8	0.061 (2)	0.049 (2)	0.054 (2)	−0.010 (2)	−0.007 (2)	0.0110 (18)
O9	0.125 (4)	0.062 (3)	0.072 (3)	−0.010 (3)	−0.032 (3)	−0.006 (2)
O10	0.039 (2)	0.054 (2)	0.059 (2)	−0.004 (2)	0.000 (2)	0.0128 (19)
O11	0.055 (3)	0.092 (4)	0.144 (4)	0.002 (3)	−0.022 (3)	0.028 (4)
O12	0.062 (3)	0.044 (2)	0.0419 (18)	0.002 (2)	−0.001 (2)	−0.0032 (16)
O13	0.086 (3)	0.071 (3)	0.088 (3)	0.003 (3)	0.034 (3)	−0.003 (2)
O1W	0.212 (15)	0.086 (8)	0.174 (12)	0.009 (10)	−0.013 (13)	−0.010 (8)
C1	0.041 (3)	0.045 (3)	0.036 (2)	0.005 (3)	0.007 (3)	0.001 (2)
C2	0.051 (3)	0.042 (3)	0.039 (2)	0.006 (3)	0.005 (3)	0.003 (2)
C3	0.062 (4)	0.052 (3)	0.034 (2)	0.003 (3)	−0.001 (3)	0.006 (2)
C4	0.061 (4)	0.039 (3)	0.041 (3)	−0.003 (3)	−0.014 (3)	0.003 (2)
C5	0.054 (3)	0.036 (3)	0.036 (2)	−0.002 (3)	0.001 (3)	−0.005 (2)
C6	0.053 (4)	0.054 (3)	0.041 (3)	−0.005 (3)	−0.008 (3)	0.004 (3)
C7	0.061 (4)	0.038 (3)	0.039 (2)	−0.001 (3)	0.000 (3)	0.004 (2)
C8	0.049 (3)	0.039 (3)	0.034 (2)	0.006 (3)	0.000 (3)	0.004 (2)
C9	0.038 (3)	0.045 (3)	0.037 (2)	0.012 (3)	0.001 (2)	0.000 (2)
C10	0.031 (3)	0.038 (3)	0.040 (2)	0.000 (2)	−0.003 (2)	0.001 (2)
C11	0.041 (3)	0.046 (3)	0.050 (3)	0.006 (3)	0.003 (3)	0.007 (2)
C12	0.047 (3)	0.053 (3)	0.040 (3)	−0.002 (3)	0.000 (3)	0.008 (2)
C13	0.052 (4)	0.051 (3)	0.041 (3)	0.000 (3)	0.003 (3)	0.006 (2)
C14	0.047 (3)	0.042 (3)	0.036 (2)	0.000 (3)	0.006 (2)	0.007 (2)
C15	0.051 (4)	0.046 (3)	0.050 (3)	0.006 (3)	0.008 (3)	0.012 (2)
C16	0.055 (4)	0.056 (3)	0.041 (3)	0.015 (3)	−0.001 (3)	0.006 (3)
C17	0.069 (4)	0.068 (4)	0.066 (4)	0.017 (4)	0.000 (4)	0.012 (3)
C18	0.091 (5)	0.063 (4)	0.044 (3)	−0.006 (4)	−0.023 (3)	−0.002 (3)
C19	0.049 (4)	0.054 (3)	0.052 (3)	0.001 (3)	−0.016 (3)	0.011 (3)
C20	0.036 (3)	0.041 (3)	0.041 (2)	0.007 (3)	0.003 (3)	−0.002 (2)
C21	0.032 (3)	0.085 (4)	0.069 (4)	0.004 (3)	0.003 (3)	0.003 (3)
C22	0.051 (4)	0.086 (5)	0.052 (3)	0.013 (4)	0.012 (3)	0.020 (3)
C23	0.072 (5)	0.096 (5)	0.099 (5)	0.018 (4)	0.035 (4)	0.026 (4)
C24	0.054 (4)	0.041 (3)	0.060 (3)	0.003 (3)	0.008 (3)	0.007 (3)
C25	0.052 (3)	0.047 (3)	0.056 (3)	0.005 (3)	0.001 (3)	−0.001 (3)
C26	0.067 (4)	0.054 (3)	0.061 (3)	0.007 (3)	0.011 (4)	0.003 (3)
C27	0.073 (5)	0.082 (5)	0.067 (4)	0.007 (4)	0.021 (4)	0.000 (4)
C28	0.081 (5)	0.085 (5)	0.076 (4)	0.023 (5)	0.016 (4)	−0.017 (4)
C29	0.082 (5)	0.057 (4)	0.107 (5)	0.023 (4)	0.005 (5)	−0.019 (4)
C30	0.085 (5)	0.047 (4)	0.096 (4)	0.003 (4)	0.016 (5)	−0.005 (3)
C31	0.086 (5)	0.065 (4)	0.073 (4)	−0.005 (5)	0.007 (4)	0.025 (4)
C32	0.102 (6)	0.079 (5)	0.095 (5)	0.002 (5)	−0.018 (5)	0.050 (4)
C33	0.071 (5)	0.052 (4)	0.077 (4)	−0.018 (4)	−0.030 (4)	0.015 (4)
C34	0.100 (6)	0.087 (5)	0.110 (5)	−0.033 (5)	−0.021 (5)	0.024 (4)
C35	0.039 (4)	0.055 (4)	0.072 (4)	0.000 (3)	−0.002 (3)	0.007 (3)
C36	0.060 (4)	0.104 (5)	0.096 (5)	−0.017 (4)	−0.010 (4)	0.024 (4)
C37	0.063 (4)	0.055 (4)	0.048 (3)	0.009 (4)	−0.001 (3)	−0.008 (3)
C38	0.073 (5)	0.047 (3)	0.045 (3)	−0.002 (4)	−0.005 (3)	−0.012 (3)
C39	0.097 (6)	0.066 (4)	0.065 (4)	−0.002 (5)	0.004 (4)	−0.008 (3)
C40	0.136 (8)	0.060 (5)	0.105 (6)	0.011 (6)	0.009 (6)	−0.017 (4)
C41	0.148 (10)	0.061 (5)	0.135 (8)	−0.020 (6)	0.022 (7)	−0.017 (5)
C42	0.103 (6)	0.071 (5)	0.124 (6)	−0.016 (5)	0.016 (6)	0.007 (5)
C43	0.077 (5)	0.054 (4)	0.085 (4)	−0.003 (4)	0.008 (4)	−0.006 (3)

### Geometric parameters (Å, °)

**Table:**

N1—C21	1.450 (7)	C14—H14	0.9800
N1—C20	1.461 (6)	C15—C16	1.487 (7)
N1—C6	1.486 (6)	C15—H15A	0.9700
O1—C22	1.334 (7)	C15—H15B	0.9700
O1—C1	1.460 (5)	C16—C17	1.322 (7)
O2—C22	1.227 (8)	C17—H17A	0.9300
O3—C24	1.338 (6)	C17—H17B	0.9300
O3—C2	1.454 (6)	C18—H18A	0.9600
O4—C24	1.203 (6)	C18—H18B	0.9600
O5—C31	1.344 (7)	C18—H18C	0.9600
O5—C3	1.464 (6)	C19—H19	0.9300
O6—C31	1.188 (8)	C20—H20	0.9800
O7—C19	1.207 (6)	C21—H21A	0.9600
O8—C33	1.388 (7)	C21—H21B	0.9600
O8—C7	1.440 (6)	C21—H21C	0.9600
O9—C33	1.174 (8)	C22—C23	1.482 (9)
O10—C35	1.320 (7)	C23—H23A	0.9600
O10—C11	1.459 (6)	C23—H23B	0.9600
O11—C35	1.207 (7)	C23—H23C	0.9600
O12—C37	1.355 (7)	C24—C25	1.484 (7)
O12—C13	1.442 (6)	C25—C26	1.366 (7)
O13—C37	1.196 (7)	C25—C30	1.413 (7)
O1W—H1A	0.8898	C26—C27	1.369 (8)
O1W—H1B	0.9036	C26—H26	0.9300
C1—C10	1.521 (6)	C27—C28	1.396 (9)
C1—C2	1.526 (6)	C27—H27	0.9300
C1—H1	0.9800	C28—C29	1.341 (9)
C2—C3	1.518 (7)	C28—H28	0.9300
C2—H2	0.9800	C29—C30	1.362 (9)
C3—C4	1.528 (7)	C29—H29	0.9300
C3—H3	0.9800	C30—H30	0.9300
C4—C19	1.518 (8)	C31—C32	1.452 (9)
C4—C18	1.530 (7)	C32—H32A	0.9600
C4—C5	1.560 (7)	C32—H32B	0.9600
C5—C6	1.526 (7)	C32—H32C	0.9600
C5—C10	1.568 (6)	C33—C34	1.492 (10)
C5—H5	0.9800	C34—H34A	0.9600
C6—C7	1.526 (7)	C34—H34B	0.9600
C6—H6	0.9800	C34—H34C	0.9600
C7—C8	1.529 (7)	C35—C36	1.444 (8)
C7—H7	0.9800	C36—H36A	0.9600
C8—C15	1.522 (6)	C36—H36B	0.9600
C8—C9	1.549 (7)	C36—H36C	0.9600
C8—C14	1.565 (7)	C37—C38	1.487 (8)
C9—C11	1.558 (7)	C38—C39	1.372 (8)
C9—C10	1.581 (6)	C38—C43	1.377 (8)
C9—H9	0.9800	C39—C40	1.418 (9)
C10—C20	1.567 (6)	C39—H39	0.9300
C11—C12	1.533 (7)	C40—C41	1.349 (12)
C11—H11	0.9800	C40—H40	0.9300
C12—C13	1.513 (8)	C41—C42	1.383 (11)
C12—C16	1.525 (7)	C41—H41	0.9300
C12—H12	0.9800	C42—C43	1.400 (9)
C13—C14	1.539 (7)	C42—H42	0.9300
C13—H13	0.9800	C43—H43	0.9300
C14—C20	1.524 (6)		
			
C21—N1—C20	118.4 (4)	C16—C17—H17A	120.0
C21—N1—C6	120.1 (4)	C16—C17—H17B	120.0
C20—N1—C6	103.9 (4)	H17A—C17—H17B	120.0
C22—O1—C1	118.8 (4)	C4—C18—H18A	109.5
C24—O3—C2	118.4 (4)	C4—C18—H18B	109.5
C31—O5—C3	119.2 (5)	H18A—C18—H18B	109.5
C33—O8—C7	115.2 (4)	C4—C18—H18C	109.5
C35—O10—C11	116.6 (4)	H18A—C18—H18C	109.5
C37—O12—C13	116.4 (4)	H18B—C18—H18C	109.5
H1A—O1W—H1B	117.6	O7—C19—C4	123.0 (5)
O1—C1—C10	103.7 (4)	O7—C19—H19	118.5
O1—C1—C2	105.7 (3)	C4—C19—H19	118.5
C10—C1—C2	115.4 (4)	N1—C20—C14	110.3 (4)
O1—C1—H1	110.5	N1—C20—C10	102.9 (4)
C10—C1—H1	110.5	C14—C20—C10	105.0 (4)
C2—C1—H1	110.5	N1—C20—H20	112.7
O3—C2—C3	111.5 (4)	C14—C20—H20	112.7
O3—C2—C1	106.8 (3)	C10—C20—H20	112.7
C3—C2—C1	110.3 (4)	N1—C21—H21A	109.5
O3—C2—H2	109.4	N1—C21—H21B	109.5
C3—C2—H2	109.4	H21A—C21—H21B	109.5
C1—C2—H2	109.4	N1—C21—H21C	109.5
O5—C3—C2	107.9 (4)	H21A—C21—H21C	109.5
O5—C3—C4	107.5 (4)	H21B—C21—H21C	109.5
C2—C3—C4	117.5 (4)	O2—C22—O1	122.4 (6)
O5—C3—H3	107.9	O2—C22—C23	126.0 (7)
C2—C3—H3	107.9	O1—C22—C23	111.7 (6)
C4—C3—H3	107.9	C22—C23—H23A	109.5
C19—C4—C3	109.3 (5)	C22—C23—H23B	109.5
C19—C4—C18	109.3 (5)	H23A—C23—H23B	109.5
C3—C4—C18	108.2 (4)	C22—C23—H23C	109.5
C19—C4—C5	113.2 (4)	H23A—C23—H23C	109.5
C3—C4—C5	109.3 (4)	H23B—C23—H23C	109.5
C18—C4—C5	107.4 (4)	O4—C24—O3	122.9 (5)
C6—C5—C4	107.9 (4)	O4—C24—C25	124.9 (5)
C6—C5—C10	99.2 (3)	O3—C24—C25	112.3 (5)
C4—C5—C10	117.3 (4)	C26—C25—C30	118.4 (6)
C6—C5—H5	110.6	C26—C25—C24	123.3 (5)
C4—C5—H5	110.6	C30—C25—C24	118.3 (5)
C10—C5—H5	110.6	C25—C26—C27	121.7 (6)
N1—C6—C5	96.3 (4)	C25—C26—H26	119.1
N1—C6—C7	112.6 (4)	C27—C26—H26	119.1
C5—C6—C7	111.5 (4)	C26—C27—C28	118.3 (6)
N1—C6—H6	111.9	C26—C27—H27	120.8
C5—C6—H6	111.9	C28—C27—H27	120.8
C7—C6—H6	111.9	C29—C28—C27	121.0 (7)
O8—C7—C6	110.7 (4)	C29—C28—H28	119.5
O8—C7—C8	109.1 (3)	C27—C28—H28	119.5
C6—C7—C8	112.1 (4)	C28—C29—C30	120.8 (6)
O8—C7—H7	108.2	C28—C29—H29	119.6
C6—C7—H7	108.2	C30—C29—H29	119.6
C8—C7—H7	108.2	C29—C30—C25	119.7 (6)
C15—C8—C7	112.9 (4)	C29—C30—H30	120.1
C15—C8—C9	114.3 (4)	C25—C30—H30	120.1
C7—C8—C9	106.9 (3)	O6—C31—O5	121.3 (7)
C15—C8—C14	112.4 (4)	O6—C31—C32	126.6 (7)
C7—C8—C14	111.7 (4)	O5—C31—C32	112.2 (7)
C9—C8—C14	97.5 (4)	C31—C32—H32A	109.5
C8—C9—C11	105.2 (4)	C31—C32—H32B	109.5
C8—C9—C10	101.3 (4)	H32A—C32—H32B	109.5
C11—C9—C10	123.4 (4)	C31—C32—H32C	109.5
C8—C9—H9	108.6	H32A—C32—H32C	109.5
C11—C9—H9	108.6	H32B—C32—H32C	109.5
C10—C9—H9	108.6	O9—C33—O8	122.9 (6)
C1—C10—C20	118.1 (4)	O9—C33—C34	126.7 (6)
C1—C10—C5	111.2 (4)	O8—C33—C34	110.4 (6)
C20—C10—C5	102.8 (4)	C33—C34—H34A	109.5
C1—C10—C9	114.3 (4)	C33—C34—H34B	109.5
C20—C10—C9	103.7 (4)	H34A—C34—H34B	109.5
C5—C10—C9	105.2 (4)	C33—C34—H34C	109.5
O10—C11—C12	110.0 (4)	H34A—C34—H34C	109.5
O10—C11—C9	113.6 (4)	H34B—C34—H34C	109.5
C12—C11—C9	111.0 (4)	O11—C35—O10	122.4 (6)
O10—C11—H11	107.3	O11—C35—C36	124.1 (6)
C12—C11—H11	107.3	O10—C35—C36	113.4 (6)
C9—C11—H11	107.3	C35—C36—H36A	109.5
C13—C12—C16	105.4 (4)	C35—C36—H36B	109.5
C13—C12—C11	111.5 (4)	H36A—C36—H36B	109.5
C16—C12—C11	106.4 (4)	C35—C36—H36C	109.5
C13—C12—H12	111.1	H36A—C36—H36C	109.5
C16—C12—H12	111.1	H36B—C36—H36C	109.5
C11—C12—H12	111.1	O13—C37—O12	122.4 (6)
O12—C13—C12	107.5 (4)	O13—C37—C38	125.2 (6)
O12—C13—C14	112.9 (4)	O12—C37—C38	112.5 (6)
C12—C13—C14	109.7 (4)	C39—C38—C43	120.2 (6)
O12—C13—H13	108.9	C39—C38—C37	117.5 (6)
C12—C13—H13	108.9	C43—C38—C37	122.2 (6)
C14—C13—H13	108.9	C38—C39—C40	119.6 (7)
C20—C14—C13	114.2 (4)	C38—C39—H39	120.2
C20—C14—C8	100.8 (3)	C40—C39—H39	120.2
C13—C14—C8	110.5 (4)	C41—C40—C39	119.1 (8)
C20—C14—H14	110.3	C41—C40—H40	120.4
C13—C14—H14	110.3	C39—C40—H40	120.4
C8—C14—H14	110.3	C40—C41—C42	122.4 (8)
C16—C15—C8	109.9 (4)	C40—C41—H41	118.8
C16—C15—H15A	109.7	C42—C41—H41	118.8
C8—C15—H15A	109.7	C41—C42—C43	118.0 (8)
C16—C15—H15B	109.7	C41—C42—H42	121.0
C8—C15—H15B	109.7	C43—C42—H42	121.0
H15A—C15—H15B	108.2	C38—C43—C42	120.6 (7)
C17—C16—C15	127.0 (6)	C38—C43—H43	119.7
C17—C16—C12	120.8 (5)	C42—C43—H43	119.7
C15—C16—C12	111.9 (4)		

### Hydrogen-bond geometry (Å, °)

**Table:**

*D*—H···*A*	*D*—H	H···*A*	*D*···*A*	*D*—H···*A*
O1W—H1A···O11^i^	0.89	2.38	3.205 (15)	155
O1W—H1B···O6	0.90	2.39	3.208 (15)	151
C15—H15A···O4^ii^	0.97	2.48	3.359 (8)	150
C23—H23B···O1W	0.96	2.58	3.491 (17)	159
C29—H29···O7^iii^	0.93	2.40	3.290 (8)	160

Symmetry codes: (i) −*x*+1/2, −*y*+1, *z*+1/2; (ii) −*x*+1, *y*+1/2, −*z*+1/2; (iii) −*x*+2, *y*−1/2, −*z*+1/2.
